# Association between dietary inflammation index and asthma COPD overlap

**DOI:** 10.1038/s41598-024-58813-1

**Published:** 2024-04-06

**Authors:** Shidong Wang, Yaokun Wang, Xiaoyan Hu, Linmin Lu

**Affiliations:** Department of Respiratory Medicine, Shaoxing Second Hospital, Shaoxing, China

**Keywords:** Asthma–COPD overlap, Dietary inflammatory index, Nutrition, NHANES, Nutrition, Public health, Diseases, Risk factors

## Abstract

There are few studies on the relationship between dietary habits and asthma–COPD overlap (ACO). In this study, we aimed to investigate the association between dietary inflammation index (DII) score and ACO. Data from the National Health and Nutrition Examination Survey (NHANES) from 1999 to 2020. The DII score was first calculated and the demographic characteristics of the grouping based on the DII quartile were assessed. The weighted logistic regression model was used to study the relationship between DII and ACO. Subgroup analysis was used to further explore the differences in different subgroups. Restricted cubic spline (RCS) plot was used to show the general trend of DII score and disease risk, and threshold effect analysis was used to determine the inflection point. In a comparison of baseline characteristics, the highest ACO prevalence was found in the fourth quartile array of people in DII. An adjusted weighted logistic regression model showed that DII was positively correlated with the incidence of ACO. Subgroup analysis showed that the association was more pronounced in women, non-Hispanics, people with cardiovascular disease, and people without diabetes. The RCS graph shows that overall, the risk of ACO increases with the increase of DII score. Threshold effect analysis showed that the inflection point was 3.779, and the risk was more significant after the DII score was greater than the inflection point value (OR 2.001, 95% CI 1.334–3.001, P < 0.001). Higher DII scores were positively associated with ACO risk. These results further support diet as an intervention strategy for ACO prevention and treatment.

## Introduction

Asthma and chronic obstructive pulmonary disease (COPD) are common diseases of respiratory system, which are caused by different etiology and show different clinical symptoms. Asthma is characterized by variable airflow restriction and airway hyperresponsiveness, causing symptoms such as dyspnea, wheezing, and chest tightness, and affects more than 300 million people worldwide^1^. COPD, often associated with smoking, causes a progressive decline in lung function and is the third leading cause of death worldwide^2^. They were previously considered to be two distinct diseases, but in recent years, it has been found that they can occur in the same patient, which we call Asthma–COPD overlap (ACO)^3^. Patients with ACO often have both characteristics, namely airway inflammation, airway hyperresponsiveness, and airflow restriction^4^. Worryingly, patients with ACO experienced more frequent exacerbations and a more rapid decline in lung function than patients with asthma and COPD alone^5^. However, the treatment plan is more simple than asthma and COPD, and other good evidence-based medical opinions are lacking except for the use of first-line inhalation preparations^6^. Therefore, early identification and intervention of ACO is very important to control disease progression.

The pathophysiological processes of ACO involve systemic inflammation and are associated with clinical manifestations, such as the impact on lung function and the frequency of acute exacerbations^7,8^. Diet can be involved in the regulation of intestinal microecology and oxidative stress response^9^, thus playing an important role in the regulation of systemic inflammation. Some nutrients with antioxidant and anti-inflammatory properties can reduce the risk of some airway diseases if consumed in the right way^10^. Therefore, dietary intervention has been used in some chronic respiratory diseases. For example, in patients with asthma, by adjusting the patient's high-fat dietary pattern, the level of systemic inflammation can be reduced, thereby preventing and relieving symptoms^11^. From this perspective, the development of a method to assess the degree of dietary inflammation in individuals is of great significance for the formulation of targeted dietary interventions.

Dietary Inflammation Index (DII) is an important parameter to evaluate the inflammatory potential of food, reflecting the degree of inflammatory diet intake in different populations. The index was developed by studying the effects of various dietary parameters on inflammatory markers, mainly considering the promotion or inhibition of inflammatory markers such as IL-6, IL-1β, TNF-α, and CRP^12^. According to the DII score, it can be judged whether the diet is easy to cause inflammation, that is, a high score means that the diet is easy to cause inflammation, while a low score means that the diet has anti-inflammatory effects^13^. This gives us a way to comprehensively assess the inflammatory potential of diet, that is, to reveal the link between diet and different diseases from an inflammatory perspective. Therefore, in recent years, DII has been gradually applied to explore the correlation between diet and chronic diseases, which has been confirmed to be associated with type 2 diabetes, cardiovascular disease, and obesity^14^. However, the relationship between DII and ACO remains unclear, so we conducted this study to explore whether inflammatory diets have an effect on ACO.

## Methods

### Study population

**Figure 1:**
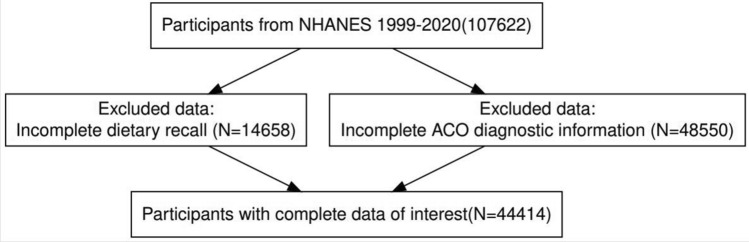
Flow chart of participants selection.

The National Health and Nutrition Examination Survey (NHANES) is a long-term national survey designed to dissect the nutrition and health status of the American population. This research protocol was approved by the Research Ethics Review Committee of the National Center for Health Statistics (NCHS). During the recruitment process, all subjects already provided written informed consent, so no additional ethical approval was required. Data for the survey was collected from 1999 to 2020. Of the 107,622 participants, we excluded 14,658 participants with incomplete or missing dietary data, followed by 48,550 participants with missing ACO diagnostic information, and finally included 44,414 participants in the study (Fig. 1).

### Calculation of dietary inflammation index (DII)

The total potential inflammatory levels of the dietary components a person consumes are added up to determine the DII^15^. The DII score was calculated using the following factors^16^: energy, protein, alcohol, beta-carotene, carbohydrate, cholesterol, total fat, fibre, folate, Fe, monounsaturated fatty acids, n-3/n-6 fatty acids, caffeine, polyunsaturated fatty acid, niacin, saturated fat, Se, vitamins A/C//E/B1/B2//B6/B12, Mg, and zinc. Firstly, Z-value is calculated for each food parameter. This is then converted to a 0-centered distribution, ranging from − 1 to + 1. Multiply that number by the inflammatory effect score for each food parameter. Finally, the values of all food parameters are added up to get the total DII score. The formula applied to the DII calculation is as follows^17^:

$$ Z\;score = (({\text{daily}}\;{\text{mean}}\;{\text{intake}} - {\text{global}}\;{\text{daily}}\;{\text{mean}}\;{\text{intake}})/{\text{standard}}\;{\text{deviation}}) $$

$$ Z\;score^{1} = Z\;score \to ({\text{converted}}\;{\text{to}}\;{\text{a}}\;{\text{percentile}}\;{\text{score}}) \times 2 - 1 $$

$$ {\text{DII}} = \sum {Z\;score^{1} \times {\text{the}}\;{\text{inflammatory}}\;{\text{effect}}\;{\text{score}}\;{\text{of}}\;{\text{each}}\;{\text{dietary}}\;{\text{component}}} $$

### Measurement of asthma–COPD overlap

The diagnosis was made according to the questionnaire and other relevant information in the NHANES database^18^. Participants were diagnosed with chronic obstructive pulmonary disease (COPD) if they met any of the following criteria: were told they had COPD; Have emphysema or chronic bronchitis and have a ratio of forced expiratory volume (FEV1) to forced vital capacity (FVC) of less than 0.7 in the first second after the application of bronchodilators. Participants were diagnosed with asthma when they met one of the following conditions: They were told they had asthma; Experienced an asthma attack. Participants with both COPD and asthma were assigned the ACO code.

### Statistical analysis

The statistical analyses were conducted using Stata/MP 17 (Stata Corp), R (version 4.2) and EmpowerStats (version 4.1). Demographic characteristics of the samples by DII quartile were assessed using chi-square and weighted logistic regression model. 404, 3593, 1977, 407, 1353, and 2984 participants were identified with missing values of educational level, family income, eosinophils number, cardiovascular disease, diabetes, and history of alcohol use, respectively. Missing values were replaced by the mean value when the variable type was continuous. For categorical variables, missing value was treated as the reference category. To investigate the linear associations between DII and ACO, weighted logistic regression model was employed. Subgroup analysis were conducted to explore the relationship between DII and ACO in different groups. The trend between DII score and ACO risk is shown by restricted cubic spline plot, and the inflection point is further analyzed by threshold effect analysis.Statistical significance was defined as a two-tailed P value < 0.05.

## Result

### Participant characteristics

Table 1 demonstrates the participant characteristics and sorted by DII quartiles. Participants in the highest DII group had a higher rate of ACO compared to those in the lowest DII group.

**Table 1:** Basic characteristics of participants by dietary inflammatory index quartile among US adults.

Characteristics	Dietary inflammatory Index				*P*-value
	Q1	Q2	Q3	Q4	
Age (years)	46.73 ± 16.36	46.85 ± 16.77	46.60 ± 17.31	46.68 ± 17.91	0.752
Gender (%)					< 0.001
Male	61.16	52.4	42.64	34.2	
Female	38.84	47.6	57.36	65.8	
Race (%)					< 0.001
Mexican American	8.37	8.69	7.86	7.07	
Non-Hispanic	80.48	79.34	80.2	81.61	
Other race	11.15	11.97	11.94	11.32	
Educational level (%)					< 0.001
≤ High school	32.19	39.39	44.51	51.27	
> High school	67.81	60.61	54.49	48.73	
Eosinophils number (1000 cells/µL)	0.20 ± 0.16	0.20 ± 0.15	0.20 ± 0.16	0.20 ± 0.17	0.009
PIR (%)	3.28	3.08	2.86	2.56	< 0.001
CVD (%)					< 0.001
No	92.74	91.19	90.65	88.05	
Yes	7.26	8.81	9.35	11.95	
Diabetes (%)					< 0.001
No	87.78	86.1	85.88	84.71	
Yes	12.22	13.9	14.12	15.29	
History of alcohol use (%)					< 0.001
No	8.71	10.49	11.63	14.12	
Yes	91.29	59.51	88.37	85.88	
ACO (%)					< 0.001
No	98.43	98.58	97.97	97.5	
Yes	1.57	1.42	2.03	2.5	

*PIR* the ratio of income to poverty, *CVD* Cardiovascular Disease, *ACO* Asthma–COPD overlap.

### Relationship between DII and ACO

The association between DII and ACO is shown in Table 2. An unadjusted model indicates a positive correlation between ACO and DII score, with each unit increase in DII score leading to a 1.131 fold increase in ACO risk. In Model 2, the association between exposure variables and outcomes was still stable after adjusting for a age, gender, and race. Model 3 shows similar results. In addition, our findings suggest that the association between higher DII and ACO is more pronounced in female populations, cardiovascular disease populations, non-diabetic populations. Subgroup analysis is shown in Table 3.

**Table 2:** Associations between dietary inflammatory index with COPD and ACO.

DII	ACO		
	OR	95% CI	P value
Model 1	1.131	1.070–1.195	< 0.001
Model 2	1.111	1.050–1.175	< 0.001
Model 3	1.076	1.017–1.139	0.011

Model 1: no covariates were adjusted. Model 2: age, gender, and race were adjusted. Model 3: age, gender, race, education level, PIR, eosinophils number, cardiovascular disease, diabetes and history of alcohol use were adjusted. PIR the ratio of income to poverty.

**Table 3:** Subgroup analysis of the association between dietary inflammatory index and ACO.

Subgroup	ACO [OR (95% CI)]	*P* value
Sex		
Male	1.031 (0.955, 1.114)	0.430
Female	1.107 (1.021, 1.201)	0.014
Race		
Mexican American	0.997 (0.805, 1.234)	0.974
Non-Hispanic	1.073 (1.009, 1.140)	0.024
Other race	1.108 (0.955, 1.285)	0.175
Education		
≤ high school	1.061 (0.988, 1.141)	0.106
> high school	1.081 (0.994, 1.175)	0.068
CVD		
Yes	1.105 (1.004, 1.216)	0.041
No	1.060 (0.989, 1.135)	0.101
Diabetes		
Yes	1.087 (0.986, 1.197)	0.092
No	1.075 (1.005, 1.151)	0.037
History of alcohol use		
Yes	1.120 (1.075, 1.167)	< 0.001
No	1.321 (1.120, 1.558)	< 0.001

### Restricted cubic spline analysis

**Figure 2:**
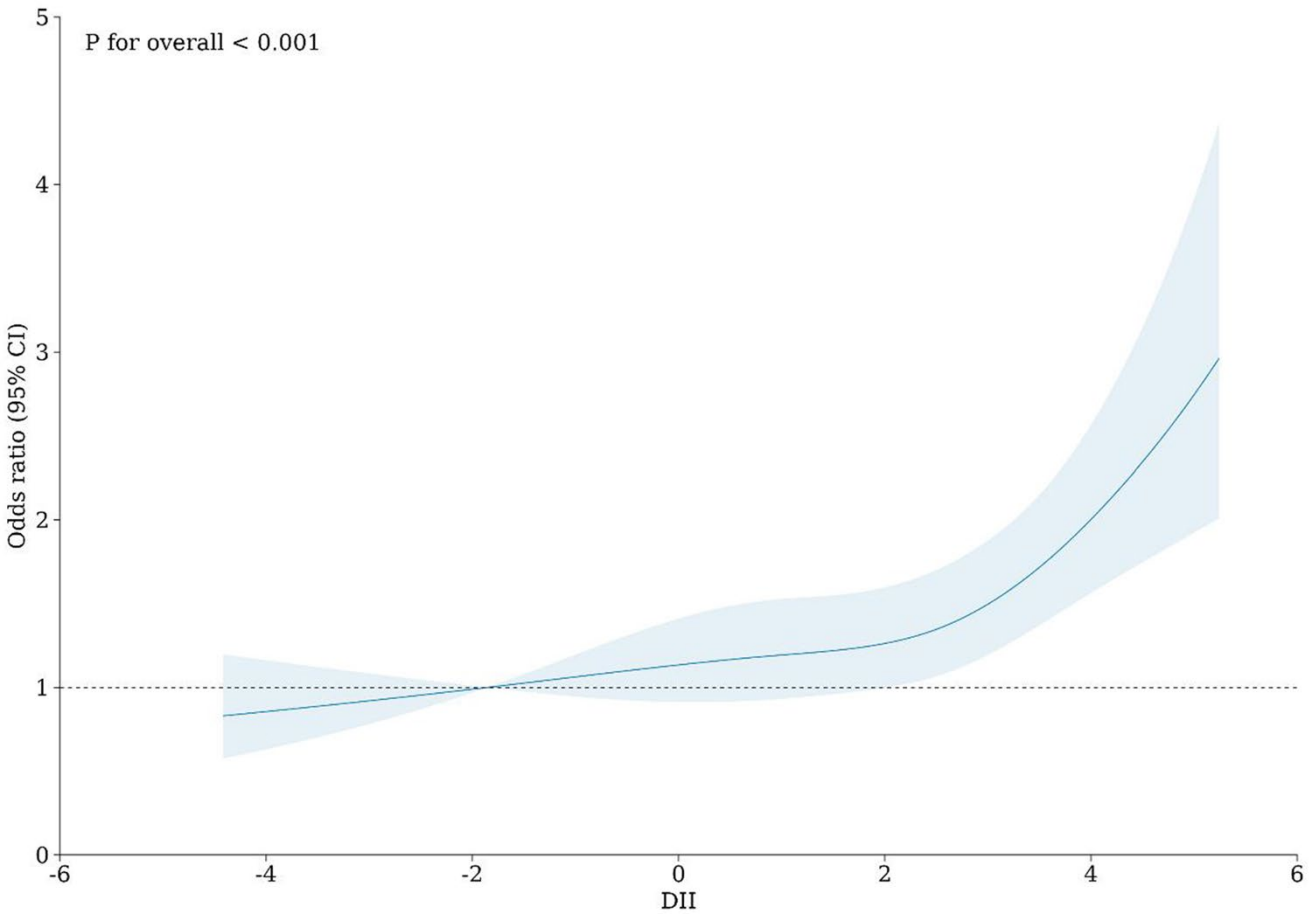
The correlation between dietary inflammation index and ACO risk.

We use a restricted cubic spline plot to visually demonstrate the relationship between DII levels and ACO. On the whole, ACO risk increases with the increase of DII scores. See Fig. 2 for the RCS diagram.

### Threshold effect analysis

Threshold effect analysis showed that the log-likelihood ratio test was significant (P < 0.001). Model II lists the break points using segmented model fitting with a break point value of 3.779. That is, when the DII score is less than 3.779, the ACO risk increases by 1.053 times for each unit increase (OR 1.053, 95% CI 1.007–1.100, P < 0.001). This risk was more pronounced after DII scores greater than 3.779 (OR 2.001, 95% CI 1.334–3.001, P < 0.001). The analysis results of threshold effects are shown in Table 4.

**Table 4:** Threshold effect analysis of Dietary Inflammation Index.

Model type	Model parameter
Model I (linear fitting)	OR (95% CI) 1.082(1.038, 1.127), P < 0.001
Model II (segmented model fitting)	
Inflection point (K)	3.779
DII < K	OR (95% CI) 1.053 (1.007, 1.100), P < 0.001
DII > K	OR (95% CI) 2.001 (1.334, 3.001), P < 0.001
Log-likelihood ratio test	P < 0.001

## Discussion

As far as we know, there is still little research on the relationship between diet and ACO. This study is the first to reveal a link between DII scores and ACO. In this observational study of 44,414 participants, we found a significant positive association between DII scores and ACO, suggesting that a higher intake of a pro-inflammatory diet may increase the risk of ACO. After adjusting the covariates, the relationship between them remains stable. And this relationship is more obvious after segmented study in threshold effect analysis. Finally, subgroup analysis of relevant stratified variables showed that this correlation could be applied to relevant populations with different gender, race, comorbidities, and alcohol use history.

The influence of diet on respiratory diseases has long been noted. For example, in patients with asthma, excessive intake of fat and high-sugar foods can easily lead to worsening symptoms^19^. In people with COPD, unhealthy diets such as processed meats, refined carbohydrates, and sweets can also increase the risk^20^. In terms of specific foods, consuming pears, apples and green leafy vegetables may help reduce the risk of COPD^21^. But previous studies have been limited to looking at a particular food and have not yet developed a scoring system to evaluate how patients eat. Tools to assess the impact of diet on ACO are still lacking. The DII index makes a comprehensive evaluation of the food intake by evaluating the anti-inflammatory characteristics of a variety of nutritional elements, which makes up for the limitations brought by the previous single study of a certain food.

This study shows that an increased DII score is associated with the risk of ACO. The DII score systematically evaluates various nutrients related to inflammation and ultimately reflects the inflammatory potential of food. A higher score indicates that the overall food intake has more nutrients that promote inflammation, and this pro-inflammatory dietary pattern is associated with increased inflammation throughout the body^22^. In patients with chronic airway inflammation, an increase in neutrophils and eosinophils in sputum can be observed after eating a high-fat meal^23,24^, which exacerbates the airway inflammatory response. Dietary fiber derived from vegetables, fruits, barley, oats and other plant foods can reduce airway inflammation in patients by down-regulating the expression of G-protein-coupled receptors 41 and 43(GPR41 and GPR43) (as indicated by FeNO and neutrophils in sputum)^25^. Some of the vitamins included in the DII calculation also play an antioxidant and anti-inflammatory role. For example, vitamin A in it can participate in the proliferation process of respiratory epithelial cells, is one of the factors that regulate lung differentiation and maturity, and also participates in the regulation of local immunity of the respiratory system to reduce inflammation^26^. Vitamin E, as a classic fat-soluble antioxidant, can regulate the production of reactive oxygen species, thereby protecting the unsaturated fatty acids in cell membranes from oxidation. In this way, it maintains the stability of the cell membrane, thereby reducing the occurrence of allergic inflammation in the respiratory system^27^. In summary, these intakes work together to determine whether the results of the dietary pattern of the participants are pro-inflammatory or anti-inflammatory, and ultimately affect the development of systemic inflammation.

Our findings are consistent with previous observations of the effects of dietary patterns on health. The advantage is that it can be evaluated by specific values, providing a further complementary explanation for the current research on the link between dietary patterns and respiratory diseases. Previous studies have often compared the traditional Western dietary pattern to the Mediterranean dietary pattern. The former is characterized by a higher intake of pro-inflammatory foods^28^, such as red meat, cured meats, refined carbohydrates, and fried foods. From the perspective of DII score, the overall score of these foods is high, which means that intake can cause an increase in the content of pro-inflammatory compounds such as oxides in the body^29^. This diet easily causes the increase of inflammatory factors such as TNF-α, CRP and IL-6, which drives the further development of airway inflammation^30^. In contrast, the Mediterranean diet is characterized by a rich intake of fruits and vegetables and moderate amounts of meat^31^. This type of diet tends to have a lower DII score, and the intake of foods with weaker pro-inflammatory properties can reduce the occurrence of respiratory inflammatory responses^32^.

There are several limitations to this study. First of all, some data of the respondents, such as lung function, CRP and other indicators, are missing in the data of some years, which makes it impossible to assess the connection between DII and them. Second, the calculation of DII is based on the recall of 24 h diet, and there may be some errors. Additionally, the definition of ACO relied solely on basic inquiries rather than comprehensive measurements, potentially diminishing the robustness of the outcomes. Finally, the data in this study came from the NHANES database, which is a cross-sectional study and needs to be validated by prospective studies.

## Conclusion

Our study shows that DII score is associated with ACO risk and can be used as a reference indicator for dietary intervention.

## Data Availability

Specific information can login web site: https://www.cdc.gov/nchs/nhanes. Anyone who meets the requirements for database usage can access the database.
